# Synergistic Activity of Ingulados Bacteria with Antibiotics against Multidrug-Resistant Pathogens

**DOI:** 10.3390/antibiotics13030200

**Published:** 2024-02-20

**Authors:** Javier Blanco-Blanco, María Bravo, Irene Simón, Pedro Fernández-Llario, Miguel Fajardo-Olivares, María Coronada Fernández-Calderón, Rosario Cerrato

**Affiliations:** 1 Ingulados, S.L., 10004 Cáceres, Spain; 2 Biosanitary Research University Institute of Extremadura (INUBE), 06080 Badajoz, Spain; koferca@unex.es; 3 Microbiology Department, University Hospital of Badajoz (HUB), 06080 Badajoz, Spain; miguel.fajardo@salud-juntaex.es; 4 Department of Biomedical Sciences, University of Extremadura, 06006 Badajoz, Spain; 5 Networking Biomedical Research Centre on Bioenineering, Biomaterials and Nanomedicine (CIBER-BBN), 28029 Madrid, Spain

**Keywords:** lactic acid bacteria, antibiotics, bacteriocins, multidrug-resistance, synergy

## Abstract

Antimicrobial resistance is a critical challenge due to the overuse of conventional antimicrobials, and alternative solutions are urgently needed. This study investigates the efficacy of compounds derived from lactic acid bacteria (LAB) fermentation combined with antibiotics against multidrug-resistant pathogens isolated from clinical cases in a hospital setting. Strains of *Escherichia coli*, *Klebsiella pneumoniae*, and *Enterococcus faecium* and *faecalis* were isolated and selected from blood, respiratory, and urine samples. They were tested against the fermentation products from the Ingulados LAB collection (BAL5, BAL6, BAL8, BAL13, and BAL16), recognized for their antimicrobial efficacy against veterinary pathogens. The activity against multidrug-resistant (MDR) pathogens was evaluated initially, followed by synergy tests using checkerboard assays and subsequent analysis. Bioinformatic assessments and supernatant treatments were performed to characterize the nature of the compounds responsible for the antimicrobial activity. Notably, BAL16 exhibited significant growth inhibition against multidrug-resistant *E. faecium*. Synergy tests highlighted its combined activity with tetracycline through FICI and surface analysis and bioinformatic analysis unveiled the protein fraction containing bacteriocins as the underlying mechanism. This study highlights BAL16 fermentation products potential as valuable antimicrobial agents against MDR *E. faecium* infections, attributed to bacteriocins. Further in-depth studies are necessary for complete bacteriocin characterization.

## 1. Introduction

Antimicrobial resistances (AMR) are among the most significant threats facing contemporary society. In 2019, an estimated 4.95 million people died from causes related to antibiotic-resistant bacteria, with 1.27 million directly caused by such pathogens. By 2050, an anticipated 10 million annual deaths worldwide are projected due to AMR-related causes, posing not only a global health threat but also an economic one. It is projected that AMR could lead to a 3.8% decrease in the global GDP by 2050 [1].

Health organizations advocate addressing this growing threat through the rational use of antimicrobials, awareness programs, employing narrow-spectrum antibiotics, prevention measures, rigorous surveillance and continuous monitoring, and adopting a ONE HEALTH approach [2]. There is also a call for increased investment in the development of new molecules with antimicrobial activity to expedite and broaden the development of new antibiotics, which is currently far below the necessary levels to address the AMR pandemic [3]. The development of novel antibiotic classes capable of overcoming existing resistance mechanisms has been impeded due to the healthcare market’s failure to appropriately recognize and remunerate the efforts in creating such products.

Since the golden age of antibiotic discovery, during which nearly all antibiotic families on the market were established, few new antibiotic structures have been discovered. This is due to the higher costs associated with developing new molecular structures com-pared to derivatives of previously known structures. Consequently, pharmaceutical companies have opted for prototype optimization over screening for new molecules with antimicrobial activity. While this approach has enhanced the spectra of activity and efficacy against multidrug-resistant pathogens, it is not sustainable to continuously modify the same structures due to an eventual limit [4].

The development of single broad-spectrum antibiotics has led to simple therapies against a wide range of pathogens, making it a highly effective, easy, and cost-efficient clinical procedure. However, this approach has inherent limitations because the selective pressure against pathogens is focused on a single mechanism. In nature, antibiotic-producing strains employ multiple and often synergistic mechanisms to compete with neighboring microbes [5].

While these policies have kept pathogens at bay, the exponential increase in AMR has prompted governments and healthcare entities to launch initiatives dedicated to correcting this situation and promoting the study of new leading structures that can reach the market and provide us with the necessary tools to tackle such pathogens. Some of these initiatives are CARB-X (Combating Antibiotic-Resistant Bacteria Biopharmaceutical Accelerator), INCATE, REPAIR Impact Fund, AMR Action Fund, or the PASTEUR (The Pioneering Antimicrobial Subscriptions To End Up surging Resistance) Act [6].

One of the strategies gaining momentum recently has been the use of host-adapted microorganisms possessing antagonistic activity against pathogens, such as lactic acid bacteria (LAB) present in the microbiota. LAB harbor various mechanisms to combat pathogens, including nutrient and niche competition, stimulation of host immunity, and production of antimicrobial compounds like bacteriocins [7].

LAB are Gram-positive, non-motile, non-spore forming, and rod- and coccus-shaped microorganism that can ferment carbohydrates, mainly producing lactic acid. Probiotic LAB exhibit interesting therapeutic properties and technological applications such as proteolytic activity, lactose and citrate fermentation, production of polysaccharides, high resistance to freezing and freeze-drying, capacity for adhesion and colonization in digestive mucosa, production of vitamins, and production of antimicrobial compounds [8].

Bacteriocins, small peptides secreted by LAB, were discovered some time ago, yet due to pharmaceutical companies’ disinterest, they have not been widely used as clinical antimicrobials. These small proteins exhibit characteristics that make them good candidates for antimicrobial development to combat the AMR problem, such as high activity against a wide range of pathogens, minimal inhibitory concentrations similar to antibiotics, and a narrow spectrum of action, making them an attractive option for developing a clinical treatment [9]. The narrow activity spectrum coupled with a low toxicity profile has led scientists to propose their use to develop different strategies to combat the increase in multidrug-resistant (MDR) microorganisms infections [10].

Furthermore, antibiotics, especially those with a wide spectrum of action, not only select MDR microorganisms in clinical settings but also impact the ecosystem when excreted. This affects diverse microorganisms in the environment, leading to the selection of microbial populations with antimicrobial resistance mechanisms. Through horizontal gene transfer, other bacteria may acquire these resistance mechanisms, facilitating the spread of antimicrobial-resistant microbes [11]. One of the clinical limitations of bacteriocins is their biodegradation as they are rapidly metabolized by proteases and other factors [12]. But this could be taken as an advantage in terms of their environmental impact, which is a risk for the development of microorganisms resistant to antibiotics. Bacteriocins could be used as a clinical drug that is rapidly metabolized, reducing the probability of enhancing MDR pathogens in the environment. Similar to conventional antibiotics, instances of resistance mechanisms against bacteriocins have been documented [13]. Nonetheless, due to their non-specific antimicrobial nature targeting specific molecules, their limited activity spectrum, and their rapid biodegradation, the occurrence of resistance is comparatively less frequent compared to antibiotics [14].

The use of bacteriocins as an alternative to traditional antibiotics has been the subject of extensive research, particularly in response to the resurgence of MDR pathogens. Studies have explored the potential of bacteriocins to substitute or enhance the action of antibiotics, potentially reducing the emergence of resistant strains. Additionally, research has focused on mobilizing the bacteriocin-producing commensal bacteria of the human microbiota to prevent bacterial infections at the external surfaces of human epithelia. These studies aim to identify specific and personalized treatments to prevent systemic disorders and curb the emergence of new resistance [15]. It is reported that bacteriocins produced by LAB from different sources are active against MDR pathogens and can act synergistically with several antibiotics against this type of pathogen to overcome resistance mechanisms [16].

This study employed a sample of LAB strains from the INGULADOS collection to investigate their antagonistic capacity against the growth of MDR pathogens. The objective was to identify microorganisms and characterize their inhibitory activity against these types of pathogens through a screening of their antimicrobial efficacy. Subsequently, assays were conducted combining the fermentation products of selected LAB strains with commonly used antibiotics to determine the synergy between them, as the presence of synergy could lead to a novel strategy for treating microbial infections, reducing the selective pressure and subsequently reducing the spread of MDR pathogens.

## 2. Results

### 2.1. Susceptibility Profile of the Clinical Bacterial Strains Selected

**Figure 1:**
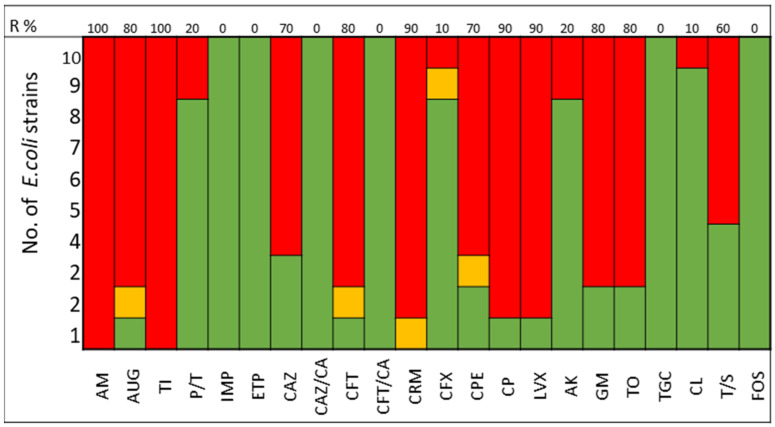
*E. coli* strains. The color green represents sensitivity to antibiotics, red represents resistance to antibiotics, and yellow represents intermediate sensitivity according to EUCAST guidelines.

**Figure 2:**
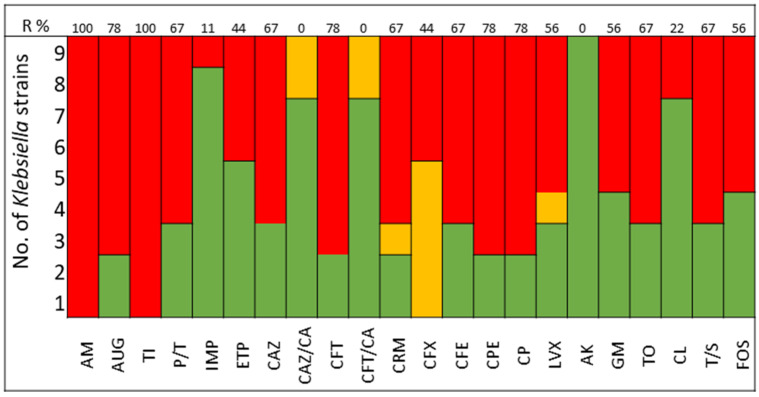
*K. pneumoniae* strains. The color green represents sensitivity to antibiotics, red represents resistance to antibiotics, and yellow represents intermediate sensitivity according to EUCAST guidelines.

**Figure 3:**
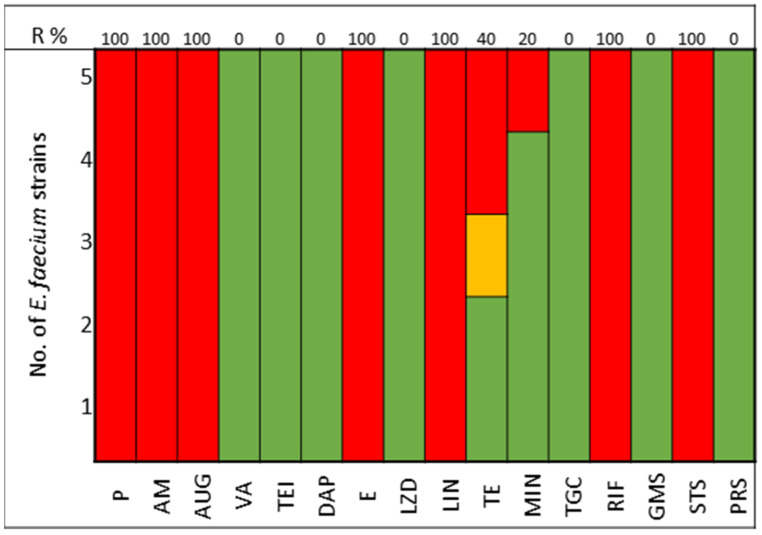
*E. faecium* strains. The color green represents sensitivity to antibiotics, red represents resistance to antibiotics, and yellow represents intermediate sensitivity according to EUCAST guidelines.

**Figure 4:**
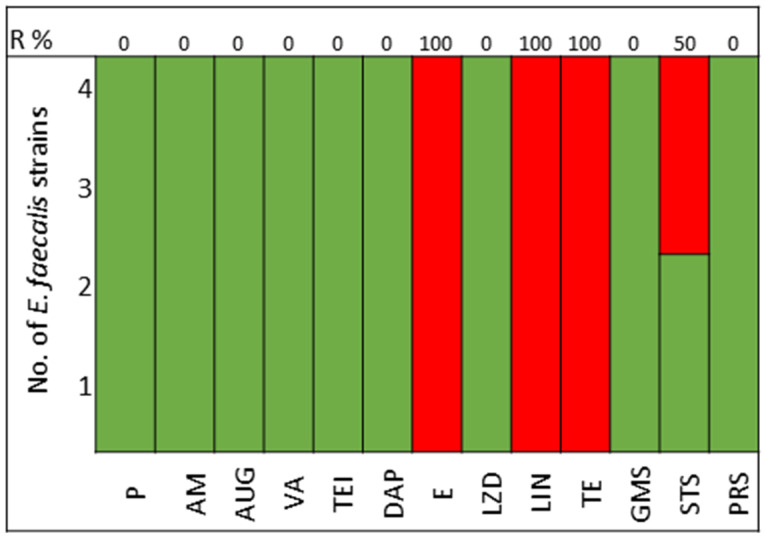
*E. faecalis* strains. Green color represents sensitive to antibiotic, red represents resistance to antibiotics.

The pathogens selected to assay the combinations between LABs Cell Free Culture Supernatant (CFCS) and antibiotics were those that presented several antimicrobials resistance mechanisms. We selected 10 *Escherichia coli* strains, 10 *Klebsiella pneumoniae* strains, 5 *Enterococcus faecium* strains, and 4 *Enterococcus faecalis* strains; the susceptibility profile of these strains of *E. coli* (Figure 1), *K. pneumoniae* (Figure 2), *E. faecium* (Figure 3), and *E. faecalis* (Figure 4), selected for the study, are presented below.

It was observed that 80% of *E. coli* strains are ESBL producers and 80% are resistant to gentamicin; all strains were susceptible to carbapenems, tigecycline, and fosfomycin (Figure 1). *K. pneumoniae* strains are ESBL producers in 50% of cases, with 60% being resistant to gentamicin and some strains exhibiting resistance to carbapenems. All strains of both *E. coli* and *K. pneumoniae* are susceptible to amikacin (Figure 2).

All strains of *E. faecium* and *E. faecalis* are susceptible to vancomycin, teicoplanin, daptomycin, linezolid, and pristinamycin and showed synergy with gentamicin. Resistance to penicillin, ampicillin, and amoxicillin-clavulanic acid is very high for *E. faecium* (100%), with all strains of *E. faecalis* being susceptible; tetracycline presents 60% of strains as either resistant or intermediate in *E. faecium*, with 100% of strains of *E. faecalis* showing resistance (Figure 3 and Figure 4).

### 2.2. Antimicrobial Assays of CFCS of the Lactic Acid Bacteria Selected

The selection of the five LAB strains from the Ingulados Laboratory collection was based on their notable antimicrobial activity against veterinary pathogens, as demonstrated in a previous study [17]. In that study more than 1500 bacterial isolates were screened for antimicrobial and immunomodulatory properties and a selection of those with interesting characteristics were included into the bacterial collection. The strains classified by their antimicrobial activities against veterinary pathogens were consequently chosen for this study to test for their antimicrobial activity against the clinical pathogen strains that were isolated at University of Badajoz Hospital Complex. Those LAB strains chosen correspond to the genera of *Lactobacillus* spp., *Enterococcus* spp. and *Lactococcus* spp. (Table 1). Using the microdilution technique, the antimicrobial effect on the selected pathogen is quantified, assessing its activity in Arbitrary Units (AU).

The activity of LABs from the Ingulados Laboratory collection varies among different strains against both Gram-negative and Gram-positive bacteria. BAL5 and BAL8 consistently exhibited antimicrobial activity against the Gram-negative bacteria *E. coli* and *K. pneumoniae*, reaching 4000 AU in both cases. Although both BAL13 and BAL16 exhibited antimicrobial activity against *Enterococcus* spp. strains, BAL16 showed the highest antimicrobial activity against these species. The observed activity against *E. faecium* was 32,000 AU, greater than that against *E. faecalis*. BAL6 showed low values against the four studied species (Table 2).

### 2.3. Single Dose Synergy Screening for Combination LAB CFCS with Antimicrobials

**Figure 5:**
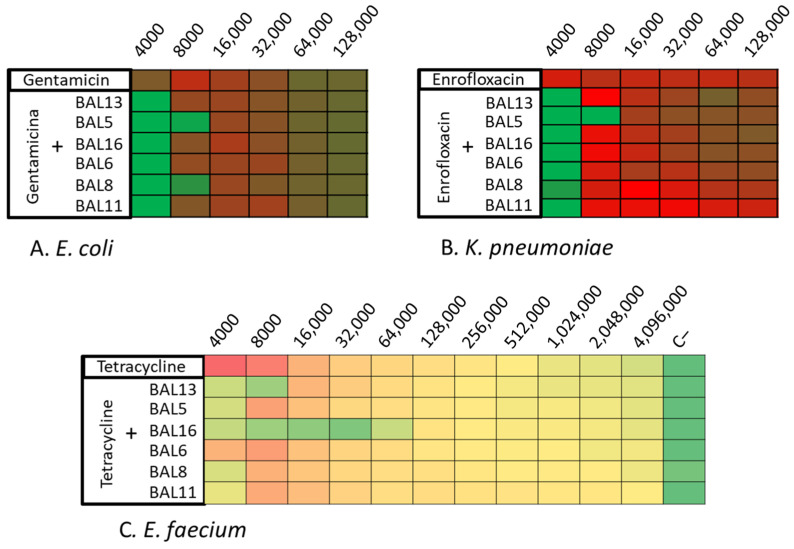
Synergy One Dose Screening of LABs CFCS combined with gentamicin, tetracycline, and enrofloxacin measured in AU. (**A**) *E. coli* + LAB CFCS + Gentamicin; (**B**) *K. pneumoniae* + LAB CFCS + Enrofloxacin; (**C**) *E. faecium* + LAB CFCS + Tetracycline. Each colored square corresponds to a well on a microtiter plate, with colors transitioning from green to yellow, and finally to red. This progression indicates bacterial growth measured by spectrometry through the use of conditional formatting based on values. In this scheme, green represents the minimum value, yellow represents intermediate values, and red corresponds to the maximum value, effectively reflecting the spectrum of bacterial growth.

Following this, these LABs CFCS were tested in combination with various antibiotics. The 28 isolates were tried with different antimicrobials combined with the LABs CFCS by microdilution assay. On their own, the CFCS of the BAL5 and BAL8 reached 4000 AU against both Gram-negative bacteria, *E. coli* and *K. pneumoniae*. Against *E. coli*, the combination of gentamicin or enrofloxacin with BAL5 and BAL8 increased the activity with one dilution, and their antimicrobial effect was increased to 8000 AU. Against *K. pneumoniae*, the most potent combination was observed between BAL5 and enrofloxacin, which reached 8000 AU. It also increased, with one dilution, the activity with other LABs tested, reaching 4000 AU (Figure 5A,B; Table 3).

On the other hand, BAL16 demonstrated a significant antimicrobial effect (32,000 AU) against *E. faecium*, and this effect did not increase when combined with the tested antibiotics. Therefore, it was chosen for further synergy studies. Additionally, in relation to enterococci, BAL13 displayed an increase in its activity when combined with certain antibiotics, ranging from 4000 to 8000 AU (Figure 5C; Table 3).

### 2.4. Effect of Treated LABs CFCS on Its Activity

**Figure 6:**
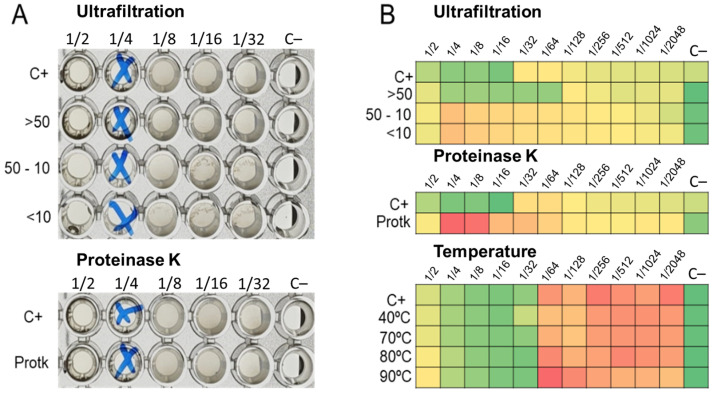
LABs CFCS antimicrobial activity after temperature treatment. (**A**) BAL5 CFCS treated and assay against *E. coli* Strain 03; (**B**) BAL16 CFCS treated and assay against *E. faecium* Strain 04. Each colored square corresponds to a well on a microtiter plate, with colors transitioning from green to yellow, and finally to red. This progression indicates bacterial growth measured by spectrometry through the use of conditional formatting based on values. In this scheme, green represents the minimum value, yellow represents intermediate values, and red corresponds to the maximum value, effectively reflecting the spectrum of bacterial growth.

**Figure 7:**
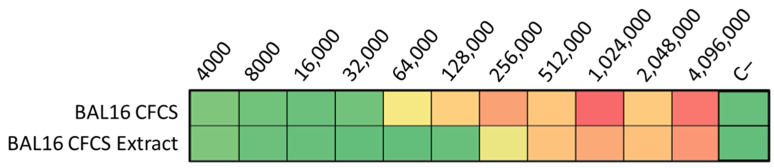
BAL16 CFCS ammonium sulphate extract antimicrobial activity against *E. faecium* Strain 04, shown in AU. Each colored square corresponds to a well on a microtiter plate, with colors transitioning from green to yellow, and finally to red. This progression indicates bacterial growth measured by spectrometry through the use of conditional formatting based on values. In this scheme, green represents the minimum value, yellow represents intermediate values, and red corresponds to the maximum value, effectively reflecting the spectrum of bacterial growth.

The ultrafiltration of LABs CFCS and subsequent activity assays against *K. pneumoniae* and *E. coli* strains revealed that the activity profiles of BAL5 and BAL8 remained unchanged, while BAL16 underwent a significant alteration. The fraction retaining its activity increased by two dilutions, rising from 32,000 AU to 128,000 AU (Figure 6). Additionally, treatment with Proteinase K led to the disappearance of antimicrobial activity in the BAL16 CFCS against *E. faecium*, in contrast to the unchanged antimicrobial activity in BAL5 and BAL8 (Figure 6). Temperature variations did not affect the activity of BAL16 CFCS, suggesting thermostability (Figure 6). Furthermore, proteinaceous compounds obtained from BAL16 CFCS through precipitation with ammonium sulfate exhibited robust antimicrobial activity compared to the original BAL16 CFCS (Figure 7).

Furthermore, proteinaceous compounds obtained from BAL16 CFCS through precipitation with ammonium sulfate exhibited a robust antimicrobial activity compared to the original BAL16 CFCS (Figure 7).

### 2.5. Bioinformatic Assay

**Figure 8:**
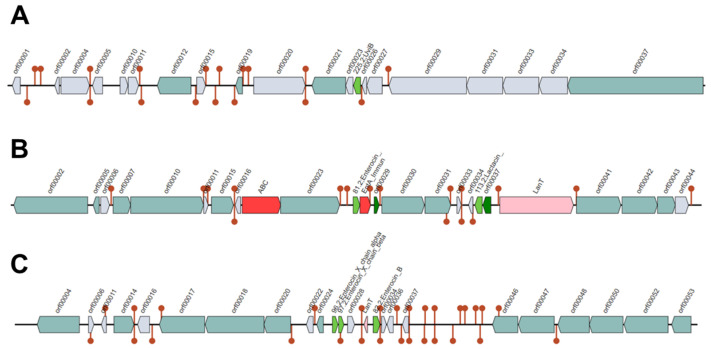
(**A**–**C**) represent three contigs containing putative bacteriocins detected by the Bagel4 program. Each bacteriocin is illustrated as a green arrow and other genetic elements are represented by different colors in the contigs such as immunity proteins. Contig (**A**) contains one putative bacteriocin, while Contig (**B**) harbors four, and Contig (**C**) features three putative bacteriocins.

**Figure 9:**
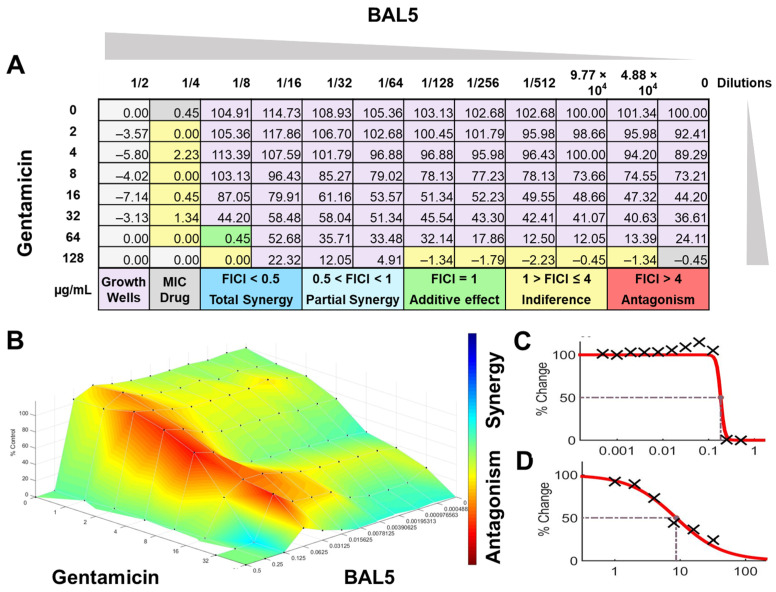
BAL5 CFCS synergy results combined with gentamicin against *E. coli* Strain 03, analyzed by FICI and surface analysis using the MIC. (**A**) FICI analysis: each cell represents a microdilution well while the numbers represent the percentage of growth; (**B**) surface analysis: the 3D curve represents the percentage of growth and the colors the synergy detected by Combenefit analysis; (**C**) BAL5 CFCS effective dose curve; (**D**) Gentamicin effective dose curve.

**Figure 10:**
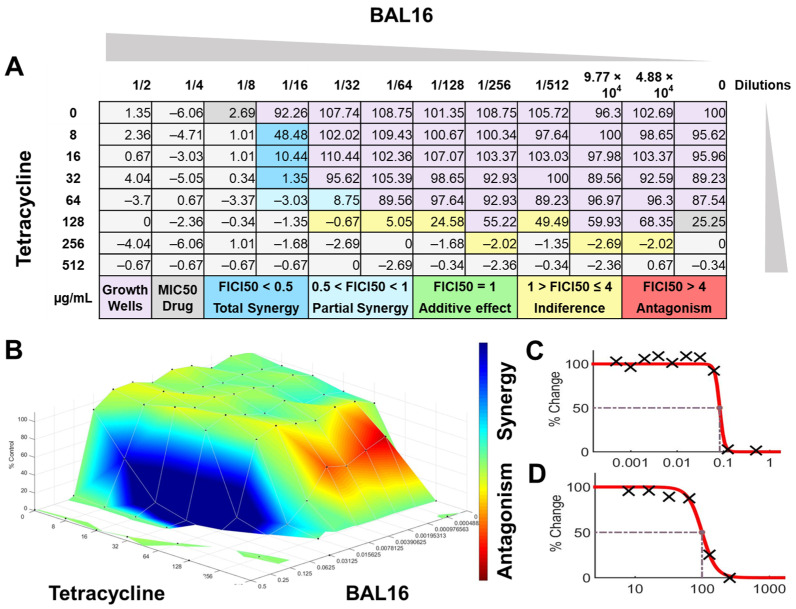
BAL16 CFCS synergy results combined with tetracycline against *E. faecium* Strain 04 analyzed by FICI and surface analysis using the CMI50. (**A**) FICI analysis: each cell represents a microdilution well while the numbers represent the percentage of growth; (**B**) surface analysis: the 3D curve represents the percentage of growth and the colors the synergy detected by Combenefit analysis; (**C**) BAL16 CFCS effective dose curve; (**D**) Tetracycline effective dose curve.

The Bagel4 analysis of the BAL18 bacterial sequence produced three contigs (Figure 8, Figure 9 and Figure 10), encompassing a total of eight distinct regions encoding putative bacteriocins.

These eight sequences associated with putative bacteriocins underwent identity percentage analysis using the NCBI protein database. The investigation unveiled a collection of bacteriocin sequences from *Enterococcus* species. The identified sequences included an EntF family bacteriocin induction factor (Sequence ID: MBC9709063.1), a Blp family class II bacteriocin (Sequence ID: WP_002374842.1), and a hypothetical protein (Sequence ID: WP_002303465.1), demonstrating 100%, 100%, and 98.72% sequence identity, respectively, with their respective reference entries.

Notably, Enterocin A (Sequence ID: EEV57261.1) and Enterocin B precursor (Sequence ID: WP_086319442.1) from Enterococcus faecium were identified with 100% sequence identity. Furthermore, Enterocin X-beta (Sequence ID: WP_086319442.1) and a partial sequence of Enterocin B (Sequence ID: ABN45881.1) from *E. faecium* exhibited 96.55% and 98.18% sequence identity, respectively. The analysis also revealed a BhlA/UviB family holin-like peptide (Sequence ID: WP_104856303.1) with 100% identity.

### 2.6. Synergy Analysis by Checkerboard Method

#### 2.6.1. Fractional Inhibitory Concentration Index (FICI)

- Antimicrobial activities of individual compounds

**Figure 11:**
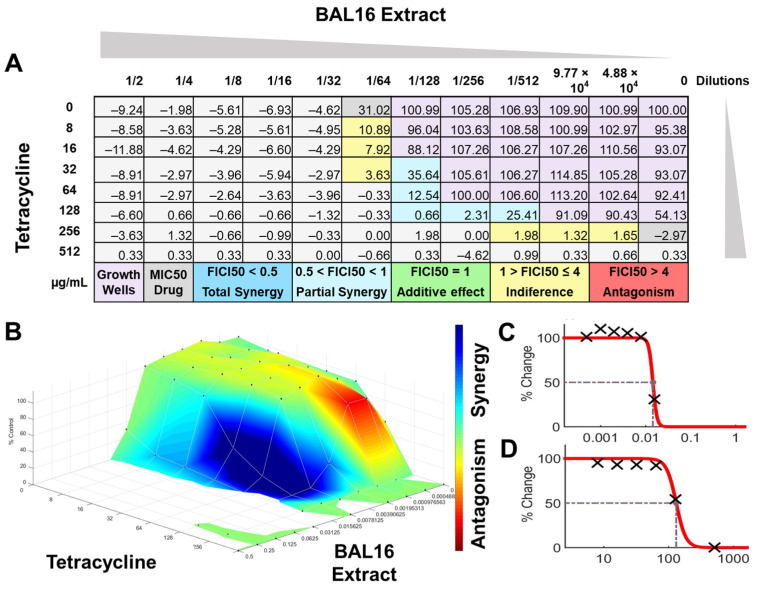
BAL16 extract synergy results analyzed by FICI and surface analysis using the CMI50. (**A**) FICI analysis: each cell represents a microdilution well while the numbers represent the percentage of growth; (**B**) surface analysis: the 3D curve represents the percentage of growth and the colors the synergy detected by Combenefit analysis; (**C**) BAL16 CFCS extract effective dose 50 curve; (**D**) Tetracycline effective dose curve.

The interactions of the LAB CFCS from BAL5, BAL8, and BAL16 with the antibiotics gentamicin, enrofloxacin, and tetracycline when they were combined together were evaluated against *K. pneumoniae* Strain 01 and Strain 02, *E. coli* Strain 03, and *E. faecium* Strain 04. BAL5 and BAL8 CFCS exhibited the same antimicrobial activities against the Gram-negative bacteria *E. coli* and *K. pneumoniae* when they were tested individually with an inhibition of 2-fold dilutions, noted as 4000 AU. The antibiotics selected, gentamicin and enrofloxacin, exhibited a Minimum Inhibitory Concentration (MIC) of 64 ug/mL and 128 ug/mL against *E. coli* Strain 03 and *K. pneumoniae* Strain 01 (Figure 9), respectively, while they exhibited 8 ug/mL and 64 ug/mL using Minimum Inhibitory Concentration 50 (MIC50). Conversely, BAL16 CFCS showed an antimicrobial activity of 8000 AU against *E. faecium* by itself, and its extract reached 32,000 AU using the MIC absolute value interpretation. And when the MIC50 interpretation was applied, the inhibition endpoint of just BAL16 extract changed from 32,000 AU to 65,000 AU. The antibiotic used to test synergy was tetracycline, and its MIC against *E. faecium* was 256 μg/mL, while its MIC50 was 128 μg/mL (Figure 10 and Figure 11).

Interpretations of the results by the FICI analysis using MIC, MIC50, and the summatory plate results obtained by Bliss synergy method (SUM-SYN-ANT) are presented in Table 4.

- FICI analysis through CMI absolute value interpretation

When the inhibition endpoint was determined by MIC, it was seen that BAL5 CFCS, in combination with gentamicin against the strain of *K. pneumoniae* sensitive to gentamicin, generated an antagonistic effect. A similar interaction was observed when the respective pathogen and antimicrobial was combined with BAL8 CFCS, although this time, FICI did not reach the determined threshold to be considered antagonistic; rather, it was considered indifferent. This indifferent effect was also observed with the same LABs against a *K. pneumoniae* Strain 01 resistant to enrofloxacin. When those LABs CFCS were combined with gentamicin against *E. coli* Strain 03 resistant to gentamicin, the effect between BAL5 and BAL8 CFCS was similar to but slightly greater that the one produced by BAL5 than BAL8. Therefore, the results obtained by FICI analysis presented both an additive and indifferent effect, respectively (Figure 9). When the BAL16 and its extract were combined with tetracycline against *E. faecium* resistant to tetracycline and the 100% inhibition endpoint was applied, the result was a partial synergy between these two antimicrobials.

- FICI analysis through MIC50 interpretation

In contrast, when using 50% inhibition of growth as an endpoint, noted as MIC50, some changes were noticed when the data were analyzed. For BAL5 and BAL8 combined with gentamicin against the *K. pneumoniae* Strain 02 sensitive to gentamicin, the results exhibited only a marginal alteration that did not change the interpretation. Also, when the results of those LABs CFCS combined with enrofloxacin against the *K. pneumoniae* Strain 01 resistant to enrofloxacin, it maintained the indifferent effect. But when the results of the checkerboard assay were analyzed using MIC50 with BAL5 and BAL8 in combination with gentamicin against *E. coli* Strain 03 resistant to gentamicin, BAL5, previously noticed as an additive effect, was interpretated as antagonism, and BAL8 continued as indifferent. Finally, both BAL16 CFCS and its extract exhibited different degrees of synergy when they were combined with tetracycline against *E. faecium* Strain 04, with the extract showing partial synergy and BAL16 CFCS displaying complete synergy (Figure 10 and Figure 11).

#### 2.6.2. Surface Analysis

The interactions analyzed by surface analysis determined by the Bliss method revealed that BAL5 and BAL8 interacted in an antagonistic manner with gentamicin and enrofloxacin against both Gram-negative pathogens as revealed by the SUM-SYN-ANT score. In contrast, the BAL16 CFCS and its extract interacted with tetracycline in a synergistic way; the SUM-SYN-ANT scores were 276 and 188, respectively (Table 4).

## 3. Discussion

### 3.1. Lactic Acid Bacteria: Properties and Therapeutic Applications

Throughout human history, the antimicrobial compounds generated by lactic acid bacteria have served as bio-preservatives in fermented foods, emphasizing the recent focus on bacteriocins [18]. These molecules are proteinaceous compounds synthesized by ribosomes and they confer to their producer an advantage in surviving in a highly competitive polymicrobial environment. They display diverse antimicrobial activity with variable spectrum and could be considered interesting candidates for further development as antimicrobial agents used in human health. The activities of bacteriocins passes from different organisms including bacteria, viruses, and fungi to structures such as biofilms. This is due to the large types of bacteriocins produced by different bacteria, offering a broad spectrum of activity to this family of molecules [19].

### 3.2. Interactions of Antimicrobial Peptides (AMPs) and Bacteriocins with Bacteria and Antibiotics

Bacteriocins are AMPs produced by microorganisms. AMPs are widely distributed in nature and can be produced by eukaryotic and procaryotic cells [20]. Despite the fact there are some differences among the molecules that make up the large group of AMPs, they share some features: a small size made of 12 to 100 amino acids (<10 KD), a positive charge, and an amphipathic structure that allows them to interact with the negatively charged bacterial membrane surface with a similar mechanism of action, mainly acting to disrupt the bacterial cell membrane [21].

**Figure 12:**
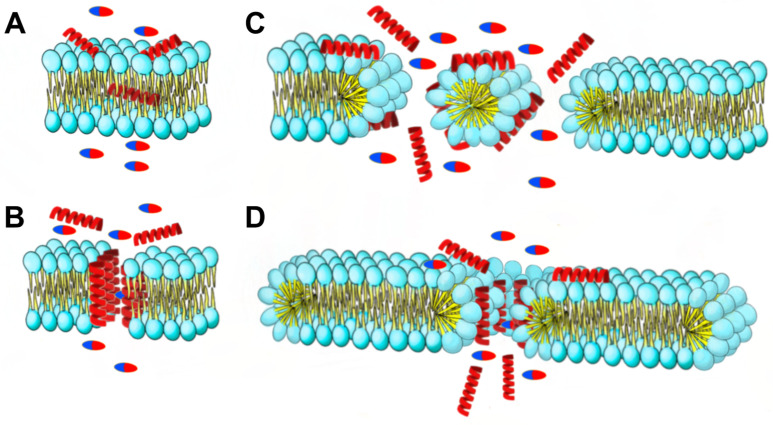
The synergy mechanisms at play between AMPs and antibiotics are multifaceted. AMPs induce changes in membrane permeability through four distinct mechanisms of destruction: (**A**) the aggregate model, (**B**) toroidal pore model, (**C**) barrel-stave model, and (**D**) carpet model. Pills: antibiotics; red helices: AMPs.

The mechanisms of action that alters the microbial membrane include pore formation or membrane degradation through the dissipation of proton motive force [22]. AMPs can create pores in bacterial cell membranes through various mechanisms (Figure 12). These include the barrel-stave model, where AMPs create holes in the membrane; the carpet model, where AMPs cover the membrane surface and form transient holes; and the toroidal pore model, where AMPs interact with the polar head of membrane lipids and form circular pores. These mechanisms differ in how AMPs interact with the cell membrane and affect its permeability. That increase in permeability is crucial to the synergism mechanism with antibiotics [23].

### 3.3. Synergies and Antagonisms between LAB Compounds and Antibiotics

In this investigation, the LAB CFCS were initially assessed individually and at a single concentration against MDR pathogens. It was found that BAL5 and BAL8 CFCS had the highest antimicrobial activity against the Gram-negative bacteria, registering 4000 AU. On the other hand, BAL18 CFCS had the greatest activity against *E. faecium* and against *E. faecalis* with 32,000 UA and 8000 UA, respectively.

Certain remarkable results were found with BAL5 and BAL8 CFCS in combination with gentamicin and enrofloxacin in the single dose screening: the activity increased from 4000 UA to 8000 UA against *K. pneumoniae* and *E. coli* suggesting a synergy between those combinations. But when those LAB CFCS were assayed by checkerboard, the results obtained and analyzed demonstrate antagonistic interactions. In addition, when the LAB CFCS of those strains were treated by ultrafiltration and proteinase K, no changes were noticed, suggesting that the antimicrobial activity was not due to the action of bacteriocins and could be a consequence of other antimicrobial compounds such a lactic acid or hydrogen peroxide [24].

On the other hand, BAL18 interacted positively with tetracycline, as was reported by the synergy analysis that took place using FICI and surface analysis. The LAB CFCS treatment with proteinase K showed that the activity was retained by the proteinaceous compounds secreted by the BAL18, and the results obtained by ultrafiltration revealed that the fraction that retained the activity was bigger than 100 KD. In addition, when proteins present at the BAL18 CFCS were precipitated by ammonium sulphate treatment, the antimicrobial activity underwent a 300% increase, and it maintained the synergy with tetracycline obtained by the checkerboard assay.

In the bioinformatic assay, it was determined that the BAL18 DNA contains sequences of eight different bacteriocins, all of them smaller than 10 KD. The comparison between the results obtained by ultrafiltration and the bioinformatic assay suggest that those proteinaceous molecules are aggregating to each other, forming bigger complexes due to their hydrophobic nature [25].

Some studies with similar approaches found synergistic interactions between antibiotics that act inside the microbial cells as macrolides, like gentamicin, with AMPs through the permeabilization of the bacterial membrane to the antibiotic [26]. In a separate investigation led by Dosler and Gerceker, the authors discovered a synergistic relationship between ciprofloxacin and nisin when employed jointly against Methicillin-resistant *Staphylococcus aureus* (MRSA) [27]. Also against this pathogen, it was found that plantaricin A, a bacteriocin produced by *Lactobacillus plantarum*, could efficiently inhibit the efflux pumps for ciprofloxacin by competitive inhibition [28]. However, as previously stated, the results of the two LAB, BAL5 and BAL8, analyzed by FICI and surface methods revealed antagonistic interactions between these LAB CFCS and the antibiotics selected. This could be due to other metabolites produced by LAB.

On the other hand, BAL18 CFCS, which did not show any relevant result on the first single dose synergy screening but encountered a great antimicrobial activity on its own, was shown to interact synergistically when it was combined with tetracycline against *E. faecium* Strain 04 by checkerboard technique and analyzed by FICI and surface analysis. As it was mentioned before, some studies with similar approaches indicate that peptides with disturbing bacterial cell membrane capabilities act synergistically with tetracycline against Gram-negative bacteria [29]. It was also found by LeBel et al. that nisin and tetracycline interact synergistically against Gram-positive *Streptococcus suis* [30].

### 3.4. Mechanisms of Action and Therapeutic Potential

Bacteriocins, the main antimicrobial compounds produced by LAB, as with most of AMPs, share a mechanism that implicates the alteration of the bacterial cell membrane by cell wall degradation or by pore formation [20]. Consequently, antibiotics and AMPs possess different mechanisms of action. While most antibiotics target specific molecules that lead to the inhibition of cellular processes, like cell wall synthesis (e.g., Penicillines) or nucleic acid transcription and replication (e.g., Aminoglycosides, quinolones and tetracyclines), most of the AMPs act in a nonspecific manner on the cell membrane. This fact turns the combination of them, AMPs and antibiotics, into a great strategy to produce two different selection pressures that could be more effective than either alone. The synergy between antibiotics and AMPs could be due to the increase permeability of the antibiotic by the AMP due to a pore formation or cell wall degradation (e.g., in the case of antimicrobials that acts on the transcription and replication). In addition, some antibiotics, such as gentamicin, produce a lipid aggregation that increases the permeabilizing capabilities of the AMP due to a major integration on the lipid bilayer, and by disturbing bacterial metabolism, which leads to a poor efficient production of enzymes synthesis and produces a synergistic activity [31].

This study reports the observed synergy between tetracycline and a proteinaceous extract sourced from a LAB CFCS. The resulting synergy is presumed to be a consequence of the bacteriocins produced during fermentation under controlled conditions, targeting MDR *E. faecium* strains isolated from clinical samples.

Not much evidence is currently available concerning the synergy of LAB CFCS or bacteriocins with antibiotics against MDR pathogens; however, a study conducted by Montalban-López et al. described that the bacteriocin AS-48 produced by the genus *Enterococcus* spp. displayed synergistic interactions with vancomycin, gentamicin, and amoxicillin/clavulanate against MDR clinical isolates [32]. It is suggested that the mechanism of action is due to the permeability increase for the antibiotic because of the effect of the AS-48, in line with what other studies have previously reported [23,26,27,29,30]. It is important to note that bacteriocins could also act by targeting specific enzymes such as the efflux pumps [28] or lipid II in the case of nisin [27].

Several approaches suggest that it is essential to conduct physicochemical studies on pathogen cell membranes and antimicrobial molecules that disrupt their integrity. This research is crucial for the development of novel antimicrobials or combinations thereof, aiming to establish effective antimicrobial therapies against MDR pathogens [33]. Continuing along this line, another synergy study conducted by Righetto and colleagues, involving a structurally modified Plantaricin 149, demonstrated the critical role of electrostatic interactions in enhancing the antimicrobial capabilities of the molecule. Through structural modifications, its effectiveness was heightened, enabling inhibition not only of Gram-positive bacteria but also Gram-negative. The study concluded that the observed synergy with vancomycin was attributed to membrane disruption mechanisms [34].

## 4. Materials and Methods

### 4.1. Clinical Bacterial Strains, Identification and Sensibility

Clinical strains isolated from different patients of a tertiary care hospital (Complejo Hospitalario Universitario de Badajoz, CHUB, Spain) were included in this study. They were collected from urinary, blood, and respiratory infections caused by the Gram-negative microorganisms *Escherichia coli* and *Klebsiella pneumoniae* and the Gram-positive microorganisms belonging to the genus *Enterococcus* spp. The strains were sent to the lab in Ingulados anonymized, in accordance with the protocol approved by the Ethical Committee of the Hospital, (Comité de Ética de la Investigación Clínica, CHUB), in order to maintain the confidentiality of the patients, and were included in the collection of microorganisms. Microorganism identification was carried out using Maldi-tof MS BioTyper (Bruker, Karlsruhe, Germany). More than 100 bacterial strains were studied, and those that presented antimicrobial resistance mechanisms against commonly used antimicrobials were chosen for further investigation.

The antimicrobial resistance was detected by MicroScan WalkAway (Beckman-Coulter, Brea, CA, USA). The following antibiotics were included in the study of Gram-negative bacteria: AM, Ampicillin; AUG, Amoxicillin Clavulanate; TI, Ticarcillin; P/T, Piperacillin tazobactam; IMP, Imipenem; ETP, Ertapenem; CAZ, Ceftazidime; CAZ/CA, Ceftazidime Clavulanate; CFT, Cefotaxime; CFT/CA, Cefotaxime Clavulanate; CRM, Cefuroxime; CFX, Cefoxitin; CPE, Cefepime; CP, Ciprofloxacin; LVX, Levofloxacin; AK, Amikacin; GM, Gentamicin; TO, Tobramycin; TGC, Tigecycline; CL, Colistin; T/S, Trimethoprim Sulfamethoxazole; FOS, Fosfomycin.

In the study of *Enterococcus* spp., sensitivity to the following antimicrobials was assessed: P, Penicillin; AM, Ampicillin; AUG, Amoxicillin Clavulanate; VA, Vancomycin; TEI, Teicoplanin; DAP, Daptomycin; E, Erythromycin; LZD, Linezolid; TE, Tetracycline; GMS, Gentamicin Synergy; STS, Streptomycin Synergy; PSR, Pristinamycin.

### 4.2. Lactic Acid Bacteria (LAB) Selection, Culture Conditions and Collection of Cell Free Culture Supernatant (CFCS)

The LAB strains used to assess their effect on the selected pathogens were BAL5, BAL6, BAL8, BAL11, BAL13, and BAL16, from the Ingulados Laboratory collection, which, in previous studies, had demonstrated antimicrobial activity against a wide range of disease-causing veterinary agents [17]. The different LAB strains were cultured in De Man–Rogosa–Sharpe (MRS) agar and the culture conditions were chosen relative to the specific strain (within 24 h–72 h and between 30 °C–37 °C). After that, a colony from each strain was then cultured in 20 mL of MRS broth to prepare a starter culture and the same culture conditions were followed. A total of 1 mL of each starter culture was then used to inoculate 100 mL of MRS broth and again, and the same culture conditions were followed.

For the collection of the CFCS, LAB cultures were centrifuged for 10 min at 5000 rpm, followed by filtration through a 0.45 µm sterile syringe filter (Labbox Labware S.L., Barcelona, Spain) to later be stored in in 15 mL centrifuge tubes at −20 °C. When needed, they were defrosted and filtered with a 0.22 µm sterile syringe filter (Branchia, Labbox Labware S.L.) before use.

### 4.3. Antimicrobial Activity Assays of LAB Supernatant by Microdilution Technique

To assess the antimicrobial capabilities of the substances secreted by LAB on clinical pathogens, the CLSI and EUCAST standards were adopted with slight modifications [35,36]. The culture media used for the antimicrobial assay were Mueller Hinton broth (MH) (Scharlab, Barcelona, Spain) and Brain Heart Infusion broth (BHI) (Biokar Diagnostics, Allone, France). Initially, the pathogens were grown on MH agar for 24 h, and then the inoculum was prepared at a 0.5 McFarland standard.

In each well of a Flat-Bottom Microplate (Deltalab, Barcelona, Spain), 50 µL of culture media broth was added. Following this, 50 µL of a different LAB CFCS were placed in each row of the first column, leaving space for both negative and positive controls. Serial dilutions were carried out to prepare antimicrobial assays for assessing the activities of LABs CFCS. The microplate was then inoculated with 50 µL of the standardized bacterial inoculum of 1 × 10^6^ CFU/mL and the plates were incubated at 37 °C overnight. The reading was performed on a microplate reader (Agilent Technologies, Santa Clara, CA, USA) at 450 nm. For the first well, if inhibition was present, it was assigned a value of 2000 AU, which was then successively doubled.

### 4.4. Single Dose Synergy Screening for Combination LAB CFCS with Antimicrobials

The combination of LAB supernatants and traditional antimicrobials was tested. The antimicrobials used for this assay included Ampicillin, Amoxicillin-Clavulanic Acid, Cefotaxime, Levofloxacin, Gentamicin, and Erythromycin. Each antimicrobial was prepared according to the recommended CLSI methods for testing minimum inhibitory concentration [35].

On a microplate containing 50 µL of culture media in each well, 25 µL of the antibiotic was added to the wells of the first column. Then, 25 µL of the six different LAB CFCS was introduced into each row of the same column. Serial dilutions were carried out to finalize the preparation of the microplate with both substances. Subsequently, the pathogen inoculum was added as described earlier and the plates were incubated.

Some strains of each pathogen were selected for the subsequent experiments: 2 *K. pneumoniae* noted as Strain 01 and Strain 02, 1 *E. coli* noted as Strain 03, and 1 *E. faecium* noted as Strain 04.

### 4.5. CFCS LABs Treatments

Different treatments were applied to the LAB CFCS to investigate the nature of the compounds with antimicrobial activity.

- Proteinase K

To each CFCS, a proteinase K solution was added to reach 0.1 mg/mL concentration, and then each CFCS was incubated for 1 h at 56 °C.

- Temperature

A total of 1 mL of each CFCS was incubated at the corresponding temperatures of 40 °C, 70 °C, 80 °C and 90 °C for 10 min using a thermoblock.

- Ultrafiltration of Supernatants

The supernatants were ultrafiltrated using Pierce™ Protein Concentrator PES (Thermo scientific, Waltham, MA, USA) with a molecular weight cutoff of 100 KD, 50 KD, and 10 KD, respectively, under manufacturer conditions. Four fractions were obtained using this method that were used to estimate the weight of the molecules responsible for the antimicrobial activity.

- Precipitation with Ammonium Sulphate

A total of 1 mL from a starter culture of LAB16 was used to inoculate 100 mL of MRS broth and culture it at 37 °C for 24 h. Afterwards, microbial cells were removed by 10 min of centrifugation at 5000 rpm at 4 °C and then filtered with a 0.22 um syringe filter. Ammonium sulphate was added to the resulting CFCS and stirred at 4 °C for 1 h. Next, the solution was centrifuged at 10,000 rpm at 4 °C for 30 min. Then, the supernatant was discarded by decantation and the pellet diluted into 10 mL of PBS. The resulting solution was filtered with a 0.22 µm syringe filter.

### 4.6. Bioinformatic Assay

The BAL16 bacterial DNA was extracted from a 24 h MRS culture at 27 °C using the E.Z.N.A. Bacterial DNA Kit (Omega Bio-Tek, Norcross, GA, USA) following the manufacturer’s instructions.

The analysis of the BAL18 bacterial genome sequence for putative bacteriocins involves leveraging the functionalities of the BAGEL4 database, a comprehensive platform designed for predicting and characterizing antimicrobial peptides. By uploading the nucleotide sequence data of the BAL18 strain onto the BAGEL4 interface in FASTA format, the database’s specialized tools for bacteriocin prediction were employed. These tools utilize algorithms and databases to scan and identify potential bacteriocins within the BAL18 sequence [37].

### 4.7. Checkerboard Method for Study of Synergistic Interactions

The synergistic interactions between the LAB CFCSs and the antibiotics chosen in this study were analyzed using the Checkerboard technique [38]. BAL8 and BAL12 were tested against *K. pneumoniae* Strain 01 and 02 and *E. coli* Strain 03, with gentamicin and enrofloxacin. BAL16 and its extract were tested against *E. faecium* Strain 04 in combination with tetracycline.

The synergy analysis was performed by two different methods: FICI and surface analysis.

#### 4.7.1. Fractional Inhibitory Concentration Index (FICI)

This method is used to determine the interaction between two compounds using the MIC of each compound alone, and in combination with the other. The formula used to calculate FICI is as follows:

$$FICI=\frac{MIC\;A\;combination}{MIC\;A\;alone}+\frac{MIC\;B\;combination}{MIC\;B\;alone}$$

The results obtained by FICI analysis are interpreted by the following measures (Table 5) [39]:

#### 4.7.2. Surface Analysis

Similar to the FICI analysis, surface analysis predicts the combined activity of two drugs using the individual dose–response curves of each compound. In this model, absorbance data is transformed into growth rate values to calculate the synergy distribution. The modelled response surface is then compared with the experimental data obtained. An important advantage of the Bliss independence model is its lack of dependence on the MIC endpoint [40]. Graphs depicting dose–response curves and surface growth using the Bliss independence method were generated using Combenefit software (Windows v2.02) [41]. The Bliss score, which indicates the percentage of synergy or antagonism, is calculated using the following formula:

$$Bliss\;Score=\left(Experimental\;Inhibition\times Expected\;inhibition\right)\times 100$$

The summatory result of the entire microplate represents a metric to evaluate synergy.

## 5. Conclusions

Conclusively, the assessment of five LAB strains showcased diverse antimicrobial activities against MDR strains of *E. coli*, *K. pneumoniae*, *E. faecalis*, and *E. faecium*. Notably, BAL16 displayed the most potent antimicrobial effect among the strains tested. Characterized by its proteinaceous nature, BAL16 exhibited synergistic activity in conjunction with tetracycline against *E. faecium*.

While further comprehensive studies are essential to fully understand the properties and mechanisms of LAB products for potential therapeutic applications, these host-adapted microorganisms produce molecules that present an opportunity to develop new therapeutic approaches for combating infections caused by MDR pathogens. Those potential therapeutic applications could be sustained by the antimicrobial discoveries highlighted here, such as developing therapies that combine these molecules with classical antibiotics to amplify their antimicrobial activity through synergistic interactions, or utilizing them independently due to their robust antimicrobial properties.

## Figures and Tables

**Table 1:** Encoding of Ingulados LABs collection.

Ingulados LAB Code	Strain
BAL13	*Lactococcus lactis* DSM 33521
BAL5	*Ligilactobacillus salivarius* CECT 9609
BAL16	*Enterococcus faecium* CECT 31026
BAL6	*Lactiplantibacillus plantarum* CECT 9608
BAL8	*Lactobacillus paracasei* CECT 9610

**Table 2:** Antimicrobial activity of CFCS Ingulados-LABs strains against clinical isolates of *E. coli*, *K. pneumoniae*, *E. faecium*, and *E. faecalis*, assessed in AU.

LAB CFCS	*E. coli* (10)	*K. pneumoniae* (9)	*E. faecium* (5)	*E. faecalis* (4)
BAL13	2000	2000	4000	2000
BAL5	4000	4000	2000	2000
BAL16	2000	2000	32,000	8000
BAL6	2000	2000	2000	2000
BAL8	4000	4000	2000	2000

**Table 3:** Synergy screening of CFCS Ingulados-LABs strains and gentamicin, tetracycline, and enrofloxacin against clinical isolates of *E. coli*, *K. pneumoniae*, *E. faecium*, and *E. faecalis*, assessed in AU.

LAB	*E. coli* (10) + Gentamicin	*K. pneumoniae* (9) + Enrofloxacin	*E. faecium* (6) + Tetracycline
BAL13	4000	4000	8000
BAL5	8000	8000	4000
BAL16	4000	4000	32,000
BAL6	4000	4000	4000
BAL8	8000	4000	4000

**Table 4:** Summary of FICI, using CMI and CMI50, and Response Surface Analysis.

Pathogen	Stain	Comp	Checkerboard Antimicrobial Activities										Response Surface Analysis	
			MIC					MIC50						
			Activity		FIC	FICI	Int	Activity		FIC	FICI	Int	∑SYN-ANT	Int
			Indiv	Comb				Indiv	Comb					
*K. pneumoniae*	01	BAL5	1/4	1/4	1	2	Indif	1/4	1/4	1	3	Indif	−592	Ant
		ENRO	128	128	1			64	128	2				
	01	BAL8	1/4	1/4	1	2	Indif	1/4	1/4	1	3	Indif	−421	Ant
		ENRO	128	128	1			64	128	2				
	02	BAL5	1/4	1/4	0.5	4.5	Ant	1/4	1/16	0.5	4.5	Ant	−341	Ant
		GEN	1	4	4			1	4	4				
	02	BAL8	1/4	1/4	0.5	2.5	Indif	1/4	1/4	0.5	2.5	Indif	−107	Ant
		GEN	1	2	2			1	2	2				
*E. coli*	03	BAL5	1/4	1/4	0.5	1	Add	1/8	1/32	0.25	4.25	Ant	−359	Ant
		GEN	64	32	0.5			8	32	4				
	03	BAL8	1/4	1/8	0.5	1.5	Indif	1/4	1/8	0.5	1.5	Indif	−742	Ant
		GEN	64	32	1			64	64	1				
*E. faecium*	04	BAL16	1/8	1/32	0.5	0.61	P.S.	1/8	1/16	0.25	0.31	T.S.	276	Synergy
		TETRA	256	64	0.125			128	8	0.06				
	04	E. BAL16	1/32	1/128	0.25	0.75	P.S.	1/32	1/128	0.25	0.56	P.S.	188	Synergy
		TETRA	256	64	0.5			128	64	0.06				

Concentrations of MIC are dilution for LAB CFCS and µg/mL for antibiotics. Abbreviatures: Comp: antimicrobial compound; Int: interpretation; Indiv: individual; Comb: combination; ∑SYN-ANT: SUM-SYN-ANT Indif: indifferent effect; Ant: antagonism; P.S.: partial synergy; T.S.: total synergy.

**Table 5:** Interpretation of the results obtained by FICI analysis.

Combination Interactions	FICI
Total Synergy	FICI ≤ 0.5
Partial Synergy	0.5 < FICI < 1
Additive effect	FICI = 1
Indifferent effect	1 < FICI ≤ 4
Antagonism	FICI > 4

## Data Availability

Data are contained within the article.
